# Urinary Tract Infection Associated With Thrombotic Microangiopathy

**DOI:** 10.5812/numonthly.12478

**Published:** 2014-01-22

**Authors:** Mohammadreza Ardalan

**Affiliations:** 1 Chronic Kidney Disease Research Center, Tabriz University of Medical Sciences, Tabriz, IR Iran

**Keywords:** Hemolytic-uremic Syndrome, Thrombosis, Urinary Tract Infections, Pyelonephritis

***Dear Editor,***

The hemolytic-uremic syndrome (HUS) is a serious disorder of microangiopathic hemolytic anemia, thrombocytopenia, and acute renal failure that is frequently associated with Shiga toxins produced in enteric infections by *Escherichia coli* O157 and *Shigella* dysenteriae, in children (1). It has also been described in non O157 *E. coli* serotypes (i.e. O104:H4). Thrombotic thrombocytopenic purpura (TTP) a closely related syndrome occurs more frequently in adults with more prominent neurological disorders (2). Both of those can be known under a common term of Thrombotic microangiopathy (TMA), and endothelial damage is the main culprit (3). Most patients with idiopathic TTP have a deficiency in the activity of ADAMTS13, that is a von Willebrand factor (vWF)-cleaving protease. It has also been suggested that UTIs could be a TTP stimulus (4). Renal endothelial cells or ADAMTS13 could be targeted by antibodies and inflammatory cytokines (5). During the last five years, we were aware about the issue, so we were able to manage some patients with this combination in our clinical center.

A 65-year old woman was admitted to our nephrology unit with malaise, fever and left flank pain. Her oral temperature was 39°C, pulse rate was 100 per minute, and blood pressure was 120/70 mmHg. Initial laboratory tests revealed leukocytosis 31000/µL, serum creatinin: 4.7 mg/dL, hemoglobin: 9.4 mg/dL, MCV: 88 fL (80-99), MCH: 27 pg (27-31), MCHC: 31 (32-36), cholesterol: 124 mg/dL, triglyceride: 232 mg/dL, platelet count: 246000/µL, serum calcium: 8.6 mg/dL, serum phosphor: 6.3 mg/dL, i-PTH: 768 pg/mL, plasma Na: 142 meq/L, plasma k: 4.9 meq/L, AST: 11 (0-31 IU/L), ALT: 10 (0-41 IU/L), alkaline phosphatase: 326 (64-306 U/L), serum iron: 26 (40-165 µg/dL), TIBC: 247 (250-450 IU/L), and serum ferritin was 180 (10-150 ng/mL for female). Urinalysis showed numerous white and red blood cells (RBC), without any dysmorphic RBC. Serum protein electrophoresis result was negative for paraproteinemia. Blood culture was positive for *E. coli* but the strain was not specified. Ultrasound examinations showed right and left kidney dimensions were 91 and 100 mm with bilateral multiple cysts. Intravenous antibiotic therapy with ciprofloxacin and imipenem was started. Patient’s general condition significantly improved and fever subsided. Platelet count dropped to 88000/µL at the third day of treatment and then dropped to 7000/µL at the sixth day of antibiotic therapy. The following laboratory data were detected; serum lactic dehydrogenase (LDH): 1150 IU/L, hemoglobin: 7.5 mg/dL, WBC: 11000/µL, reticulocyte count: 4 (0.2-2/per high power field), total serum bilirubin: 2.3 mg/dL, direct billirubin; 0.5 mg/dL and serum creatinine rose to 6.2 mg/dL, D-dimer test was negative, serum ADAMTS 13 activity was 47%. Pipheral blood smear was positive for fragmented RBC (schistocyte). With the primary diagnosis of TMA, plasma exchange with fresh frozen plasma was started (1.5 L/d). After four days, platelet count rose to 105000/µL, LDH dropped to 580 U/L, and serum creatinine level returned to 4.0 mg/dL. During all those therapeutic periods, intensive antibiotic therapy was also continued in parallel with plasma exchange.

Antibiotic therapy could increase the risk of full-blown HUS in children with STEC enteric infection (6). We observed the same situation in a patient with urinary tract infection and *E. coli* bacteremia. However this should not discourage the administration of appropriate antimicrobial agents in this situation, as it may lead to death (7). We found out, from this patient and from other patients who managed the same condition in our unit (author’s unpublished data), that a parallel combination of antibiotic and plasma exchange should be implemented in this condition. In an adult patient with pyelonephritis a superimposed TMA could be a reason of renal function deterioration. This situation might be more common and less considered in the developing countries.
